# Plain Radiography for Diagnosing and Monitoring Foot Osteomyelitis in Persons With Diabetes: Accuracy, Limitations and Clinical Utility

**DOI:** 10.1111/wrr.70128

**Published:** 2026-01-23

**Authors:** Henry S. G. Harrison, Edgar J. G. Peters, Mario Maas, Lawrence A. Lavery, Kerensa M. Beekman

**Affiliations:** ^1^ Amsterdam, Section of Infectious Diseases, Department of Internal Medicine Amsterdam UMC, Vrije Universiteit Amsterdam the Netherlands; ^2^ Vrije Universiteit and the AMC Amsterdam Amsterdam the Netherlands; ^3^ Amsterdam Movement Sciences, Rehabilitation and Development Amsterdam the Netherlands; ^4^ Amsterdam Infection & Immunity, Infectious Diseases Amsterdam the Netherlands; ^5^ Amsterdam UMC, AMC Amsterdam, Department of Radiology and Nuclear Medicine Amsterdam the Netherlands; ^6^ Amsterdam Gastroenterology Endocrinology Metabolism Amsterdam the Netherlands; ^7^ Department of Orthopedic Surgery University of Texas Health Science Center at San Antonio San Antonio Texas USA

**Keywords:** diabetic complications, diabetic osteomyelitis, diagnostic accuracy, monitoring, plain radiography, serial radiography

## Abstract

Osteomyelitis of the feet is common in persons living with diabetes due to peripheral artery disease, peripheral neuropathy, and increased susceptibility to infection. Although plain radiography is a low‐cost and widely available diagnostic tool, its diagnostic performance is limited. Serial radiography may improve the accuracy and clinical utility. This systematic review studies the diagnostic accuracy, limitations, and clinical utility of singular versus serial plain radiography for diagnosing osteomyelitis in the foot in persons with diabetes at diagnosis and follow‐up. We conducted PubMed and Embase searches for articles on the diagnostic performance of serial plain radiography for osteomyelitis of the foot in patients with diabetes. Multiple *z*‐tests were used to compare the performance of singular and serial radiographs. Fourteen studies were included, with only one providing original data on serial radiography. The sensitivity of singular radiography ranged from 22% to 93%, and specificity ranged from 22% to 94%. Serial radiography had a sensitivity of 89% and a specificity of 38%. Of the 13 studies, serial radiography outperformed singular radiography in terms of sensitivity in three reports but failed to outperform singular radiography on specificity in any of the reports. The initial examination indicated little advantage of serial radiography over singular radiography for the diagnosis of diabetic foot osteomyelitis. However, a significant exclusion bias exists due to the lack of research in this area. Further research is warranted to clarify the clinical utility of serial radiography.

**Abbreviations**
CRP: C‐reactive protein
CT: computed tomography
ESR: erythrocyte sedimentation rate
HbA1c: haemoglobin A1c
MRI: magnetic resonance imaging
PS: prospective study
RS: retrospective study
SD: standard deviation
WBC: white blood cell count
X‐ray: X‐radiation (plain radiography)

## 1 Introduction

Osteomyelitis of the foot is an infection of the bone and the bone marrow distal to the malleolus [1, 2, 3]. Bone infarction, arthropathy, sepsis, and amputation are common complications, especially in the absence of treatment [4]. Early diagnosis and treatment are essential factors when treating osteomyelitis, as this can significantly reduce severe complications, in addition to highly invasive interventions such as amputation [5]. Osteomyelitis can occur either haematogenously or exogenously [6]. In the foot, the route of infection is predominantly exogenous, occurring through skin lesions or ulcers. These are common in persons with diabetes. Typical clinical signs of osteomyelitis include local inflammation, tissue damage, and palpable bone in the ulcer base [7]. Patients with diabetes, particularly those with neuropathic foot ulcers, may present with redness, swelling, and warmth, along with systemic symptoms such as fever [4, 5, 8, 9]. Timely diagnosis and subsequent monitoring of osteomyelitis are essential to initiate appropriate treatment and evaluate its effectiveness as well as the extent of damage to the bone and surrounding structures [5].

Osteomyelitis is predominant in patients aged < 20 years and > 50 years. Its risk factors include poor tissue perfusion, tissue injury, and open fractures, which can be exacerbated by immunosuppression, intravenous drug use, and systemic diseases [5, 10, 11]. In this review, we focused on patients with diabetes and osteomyelitis of the foot via an exogenous route. The incidence of osteomyelitis of the foot has increased substantially with an increase in global diabetes rates [6]. This patient group is particularly relevant because of its high disease prevalence in the global population (14%) and the combination of risk factors, including peripheral neuropathy, peripheral artery disease, and susceptibility to infection (immunopathy), which make this patient population particularly susceptible to osteomyelitis of the foot, where up to 50%–60% of sever diabetic foot ulcers result in amputation [12]. Furthermore, osteomyelitis worsens recovery owing to reduced healing capabilities in persons with diabetes [13].

Bone biopsy is the diagnostic reference standard for osteomyelitis [8] and can be supported by clinical presentation, physical examination including a probe‐to‐bone test, laboratory assessment such as white blood count (WBC), erythrocyte sedimentation rate (ESR), C‐reactive protein (CRP) level, and imaging, of which plain radiography (dual‐energy) CT, and MRI are the most common; nuclear medicine techniques, particularly labelled white blood cell (leukocyte) scintigraphy (often combined with Single Photon Emission Computed Tomography/CT) are also used as comparator modalities in diabetic foot infection work‐ups and are referenced in several of the included studies. However, these advanced imaging techniques such as (Dual‐energy) CT and MRI offer higher sensitivity but are limited in availability [7, 8, 14, 15, 16]. Bone biopsies are invasive, potentially painful, and distressing, often requiring local anaesthesia and training, and a prolonged waiting time of several days for culture to confirm the diagnosis. Imaging can help improve diagnosis, avoid unnecessary invasive tests, and rule out other differential diagnoses [5, 17].

Plain radiography is by far the cheapest imaging modality and is easy to perform [18]. In routine practice, plain radiography also serves a first‐line screening and ‘gatekeeping’ role by helping identify alternative diagnoses confounders (e.g., Charcot neuro‐osteoarthropathy, fracture or foreign bodies) and in doing so, informing escalation to MRI, CT, or nuclear imaging where suspicion remains high. However, plain radiography's clinical diagnostic utility is highly dependent on the stage and severity of the disease, where it has less sensitivity for detection and monitoring in the early stages of the disease. This is a consequence of the delayed corresponding changes associated with osteomyelitis (bone destruction, periosteal changes, and demineralisation) materialising later on in the disease [9, 14, 19]. Due to these delayed changes that are visible on plain radiography, it is plausible that repeated radiography could deliver significantly increased diagnostic and monitoring values over a single plain radiograph, as the subsequent radiograph will capture the disease as a later and more advanced stage of bone pathology, as well as allowing a repeated opportunity for radiological examination; this logic marks the basis of the Hingorani et al.'s [20] guidelines for the management of the diabetic foot. However, little is known about the real‐world performance of serial plain radiography in diagnosing and monitoring disease progression, how it compares to the performance characteristics of singular plain radiography and other imaging modalities, or its subsequent clinical utility in practice.

In this review, we aimed to evaluate the diagnostic accuracy, limitations, and clinical utility of serial plain radiography in diagnosing and monitoring diabetic osteomyelitis of the foot compared with a single radiograph.

## 2 Materials and Methods

The PubMed and Embase electronic databases were selected for the literature search. PubMed was selected for its comprehensive coverage of biomedical and health‐related research, and Embase to improve retrieval of radiology and nuclear medicine literature through complementary indexing (Emtree) and broader European journal coverage. Together, these databases provide broad and complementary coverage of the relevant scientific literature. Given the focused nature of the research question and substantial overlap with other bibliographic databases, the use of these two databases was considered sufficient to identify the relevant literature.

**FIGURE 1:**
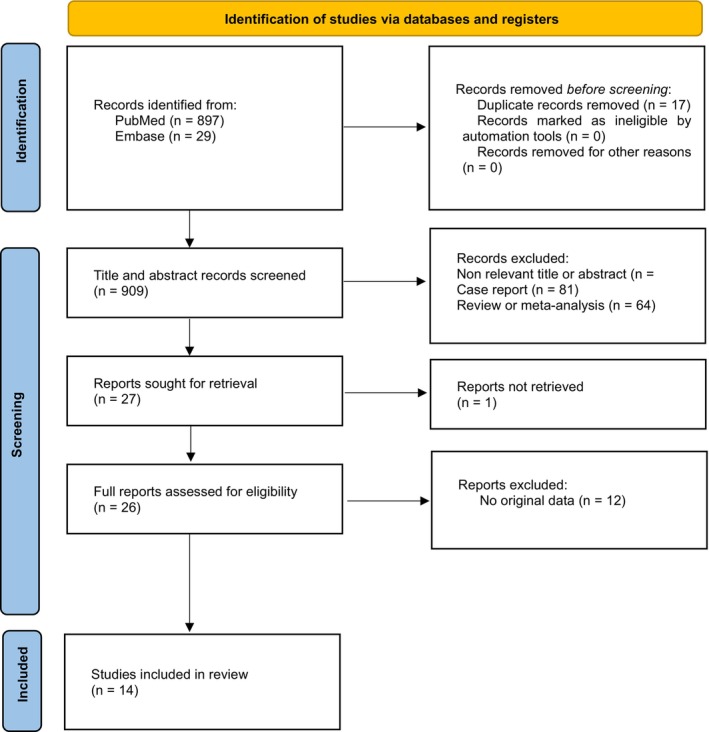
Selection flow diagram.

The search strategy included terms related to diabetic osteomyelitis and plain radiography. The following was the final search term used for PubMed: (“Plain radiography” OR X‐ray OR Radiography OR Xray OR serial radiography OR serial radiograph) [Title/Abstract] AND (diabetic foot osteomyelitis OR Diabetic bone infection OR diabetic foot bone infection) [Title/Abstract] AND (“accuracy” OR “diagnosis” OR “monitoring” OR “clinical utility” OR limitations OR follow up OR follow‐up). And for Embase the following search term was used: (‘Plain radiography’:ti,ab OR ‘X‐ray’:ti,ab OR ‘Radiography’:ti,ab OR ‘Xray’:ti,ab OR ‘serial radiography’:ti,ab OR ‘serial radiograph’:ti,ab) AND (‘diabetic foot osteomyelitis’:ti,ab OR ‘Diabetic bone infection’:ti,ab OR ‘diabetic foot bone infection’:ti,ab) AND (‘accuracy’:ti,ab OR ‘diagnosis’:ti,ab OR ‘monitoring’:ti,ab OR ‘clinical utility’:ti,ab OR ‘limitations’:ti,ab OR ‘follow up’:ti,ab OR ‘follow‐up’:ti,ab) The search was conducted on the 15th of July 2025; no language or date limitations were applied. A search of clinicaltrials.gov was conducted using the terms serial radiography and diabetic foot osteomyelitis to assess publication bias.

Studies that included empirical diagnostic performance metrics for both singular and serial plain radiography for the detection and diagnosis of diabetic osteomyelitis of the foot were included. Studies were excluded if they were case reports, animal studies, or if they lacked quantitative performance characteristics. One reviewer (H. S. G. H.) retrieved the articles and assessed their eligibility based on the inclusion and exclusion criteria.

Rayyan.ai was used for abstract screening and duplicate removal, and the articles were filtered, manually read, and assessed for eligibility. Initial screening was conducted based on the title and abstract, and eligible articles were retrieved and assessed for eligibility.

Data extraction was performed, and the following data were collected if available:

– Study characteristics: year of publication, country of origin, study design, number of radiologists, and years of experience.
– Participant characteristics included sample size, demographics, sex distribution, age, infectious organisms, disease stage, diabetes status, and location of osteomyelitis.
– Outcomes: sensitivity and specificity.

Studies that investigated singular plain radiography were compared in terms of their sensitivity and specificity to serial plain radiography, using multiple *z*‐tests for proportions. Due to the risk of multiple *z*‐tests increasing family‐wise type 1 errors, a Bonferroni correction was applied to the selected 0.05 significance level.

A risk of bias analysis was conducted on selected articles using QUADAS‐2 methodology [21].

## 3 Results

Of the 909 articles identified after duplicate removal, 27 were retrieved for eligibility assessment (Figure 1). One Embase article could not be retrieved (To study the various Clinical and Radiological Presentation of patients with Diabetic foot Osteomyelitis at Tertiary care centre. DOI: 10.31838/jcdr.2023.14.04.334). A total of 14 articles were included: 13 articles investigated singular radiography and only one article investigated serial radiographs in the diagnosis of diabetic osteomyelitis. In that study, patients were included if serial plain radiography was conducted between 14 and 35 days after the initial radiograph, with an average interval of 29 days (SD 6.6) between radiographs [22]. An overview of the extracted data is presented in Table A1.

In searching clinicaltrials.gov no relevant trials were found.

In all studies, it was possible to retrieve complete data on the following characteristics: author, publication year, country of study, design, sample size, sensitivity, and specificity. Eleven studies were conducted before 2000, with only one published within the last 10 years (2022). Eight studies were conducted in the USA, one in Canada, and four in Europe. The age of the participants was not reported in three studies (see Table A1). In five studies, the proportion of male to female patients was not reported, but in those that did, the majority were male, ranging from 44% to 96%. Eight studies did not contain data on the average duration of diabetes before osteomyelitis diagnosis; however, the mean age of diabetes duration in the participants ranged from 11 to 22 years. Leone et al. [22] and Oyen et al. [33] reported the proportion of patients with type 2 diabetes as 90% and 81%, respectively. Three studies, Leone et al. [22], Morales et al. [24], and Levine et al. [25] reported the location of osteomyelitis in the forefoot (82%, 98% and 92%, respectively), midfoot (7%, 1%, and 0%, respectively), and hindfoot (11%, 1% and 8%, respectively). Only Leone et al. reported culture results from bone biopsy (conducted on 26 of the 46 patients) and found gram‐positive bacteria in 86% and gram‐negative bacteria in 15% of the cases. Only four studies reported the number of radiologists who evaluated the images, ranging from one to four evaluators, of which only one study reported the years of experience of the radiologists. Inter‐rater variability was not reported in any of these studies. An analysis of whether experience correlated with higher sensitivity and specificity was not possible due to the sparsity of the data available.

Sensitivity and specificity were reported in all studies. In singular plain radiography, the sensitivity ranged from 22% to 93% and the specificity ranged from 22% to 94% compared with several different reference standards (Table A1). For serial radiography, sensitivity and specificity were 89% and 38%, respectively. Multiple *z*‐tests were conducted to compare the sensitivity and specificity between the individual singular plain radiography tests and Leone et al.'s serial plain radiography sensitivity and specificity.

The Bonferroni correction was applied to the significance level.

$$a_{\text{corrected}}=\frac{0.05}{13\times 2}\approx 0.00192$$

The *z*‐test for the proportions was executed between Leone et al.'s sensitivity and specificity and the single radiography characteristics.

$$Z=\frac{p_{1}-p_{2}}{\sqrt{p\left(1-p\right)\left(\frac{1}{n_{1}}+\frac{1}{n_{2}}\right)}}$$

Table A3 presents the results of this analysis.

The results indicated that the sensitivity of serial radiography was higher than that of singular plain radiography in three of the 13 studies. However, the specificity of serial radiography did not surpass that of singular radiography in any of these 13 studies. Furthermore, in three studies, singular radiography outperformed serial radiography in terms of specificity. These results indicate that there appears to be limited additional diagnostic value of serial radiography, and that the test characteristics of serial radiography rarely outperform singular radiography for the identification of osteomyelitis.

Leone et al. [22] conducted the only study that investigated repeated plain radiography for the detection and diagnosis of foot osteomyelitis in persons with diabetes. In their study, a retrospective single‐institution study was performed, which included patients with a non‐specified clinical suspicion of tissue and bone infections who had serial plain radiographs taken between 14 and 35 days after the initial radiograph. A subsequent T1 weighted MRI with fluid‐sensitive and gadolinium‐enhanced sequences, performed within 2 weeks of radiography, served as the reference standard. In this study, plain radiographs of the lateral, anterior posterior, and medial oblique were obtained and the parameters of periosteal reaction, osteopenia, presence of gas in the surrounding soft tissues, and signs of bone destruction were evaluated. Two radiologists with 3 and 30 years of experience in musculoskeletal radiography evaluated the images to a consensus. They found that serial plain radiography demonstrated an overall sensitivity of 89% and a specificity of 38%. Among the radiographic features, bone destruction was the most reliable diagnostic sign, with a sensitivity of 89% and a specificity of 88%. In contrast, other findings showed substantially lower diagnostic accuracy: osteopenia (sensitivity 32%, specificity 50%), gas in the soft tissues (sensitivity 18%, specificity 60%), and periosteal reaction (sensitivity 11%, specificity 34%).

The risk of bias analysis included 13 studies that were analysed using QUADAS‐2 methodology shown in Table A2. Patient selection was rated as low risk of bias in four of the 13 studies, unclear in seven, and high in two. The high or unclear risk was primarily due to the retrospective design and poorly described inclusion criteria. The reference standard bias was low across all studies, with most studies using bone biopsies. Index test bias was unclear in 62% of the studies due to the lack of blinding and the absence of pre‐specified thresholds. Flow and timing were also unclear in 30% of the cases, often due to missing information or details. Applicability concerns were low for both the reference standards and applicability. However, the patient selection application raised moderate or high concerns in eight studies due to selective or highly specific study populations.

## 4 Discussion

This systematic review assessed the performance of serial plain radiography versus singular plain radiography in patients with diabetic foot osteomyelitis. Serial radiography demonstrated higher sensitivity in some studies; however, it had lower specificity. The results of one serial radiography study showed that serial plain radiography did not meaningfully outperform singular plain radiography.

The QUADAS‐2 assessment revealed notable variations in study quality across key domains. While reference standards were consistently appropriate, many studies showed an unclear or high risk of bias in patient selection and index test interpretation. Retrospective designs and poorly reported inclusion methods are common, which increases the risk of spectrum bias. Blinding and the use of predefined diagnostic thresholds have often been inadequately reported, especially in earlier studies. Flow and timing were frequently unclear because of missing details regarding the test intervals or exclusions. Applicability was generally good for index tests and reference standards, but nearly half of the studies had moderate concern for patient selection, often enrolling highly specific or severe cases from tertiary centres. Studies with low risk across all domains, such as Blume et al. [35] and Lipman et al. [31], employed prospective designs, blinding, and clear test protocols, highlighting key methodological features for future research. Improving the study design, standardised reporting, and ensuring real‐world applicability remain essential to strengthen diagnostic evidence for foot osteomyelitis in people with diabetes.

The extremely limited study pool and inconsistency in standardisation of study design and reporting, as well as the lack of recent modern studies, highlight the gaps in the current evidence. Furthermore, no studies were identified that evaluated the use of plain radiography in the monitoring of diabetic foot osteomyelitis, leaving this as an unexplored potential benefit of serial radiography.

Studies on singular plain radiography have shown considerable variability and lack of homogeneity in study characteristics, and consistency in reporting from one author to another. The small sample sizes in the majority of studies also contributed to a high likelihood of imprecision in the reported imaging performance, where their reported performance characteristics varied significantly, with a sensitivity of 22%–93% and a specificity of 22%–94%. In addition to the small sample size, this variability could be attributed to multiple factors, including variability in patient populations (e.g., age, sex, disease duration, diabetes type, and glycated HbA1c). This variability is also consistent with the known limitations of plain radiography, namely, that there is significantly reduced sensitivity and specificity in the early stages of the disease owing to the delayed radiographic changes that are visible [9, 14, 19]. Furthermore, the performance of radiography depends on the skill and experience of the operator [36]. In these studies, the characteristics of the radiographic evaluator were not consistently reported; however, it is likely that the skill and experience differed significantly among the studies. Another shortcoming was the lack of standardised criteria for the diagnosis of radiographic osteomyelitis or the reporting thereof, leading to uncertainty in the pathological signs used for diagnosis. Therefore, the performance of plain radiography in these studies is highly reliant on the stage of the disease and the operator. However, these factors cannot be accounted for as confounders as they have not been consistently reported. In addition to the technical parameters of the imaging, diagnostic accuracy is influenced by the experience and skill of the radiographer. Radiographer technique can impact image quality, while radiologist experience affects the image quality and readability. Future studies should consider measuring radiograph quality and recording radiographer experience in order to stratify diagnostic performance on these factors. Furthermore, reference standards from MRI to bone biopsy, as well as the radiographic views employed and machines used, differed greatly or were not reported at all, further contributing to the difficulty in providing a consistent comparable standard between studies. Future studies should control, report, and standardise these factors.

This study aimed to examine both the diagnostic value and the value of plain serial radiography for disease monitoring. In this review, the utility of serial radiography for monitoring was unclear, as in all studies, the focus was on diagnostic performance compared with a reference standard. Radiographic monitoring could provide important insights into the effectiveness of treatment and disease resolution and offer a low‐cost accessible means to track this. Therefore, the value of serial plain radiography in monitoring disease progression warrants further investigation.

This study has several limitations that deserve consideration. Regarding the analysis, the Bonferroni correction for significance is conservative and could lead to more type 2 errors. Only one singular study examined serial radiography, and as we observed that its diagnostic value varied widely, it is plausible that serial radiography would experience similar variability. Furthermore, most studies were conducted before 2000, and there have been meaningful developments in imaging techniques that may impact the value of this imaging modality. Additionally, all studies lacked standardisation in the reporting of their findings regarding the reporting of the diagnosis, the data they were tracking, and the indications for diagnoses; standardisation of this aspect could increase reliability and reportability of studies.

These findings indicate that serial radiography did not have a higher sensitivity or specificity than those found in the majority of studies investigating singular plain radiography. Future studies should focus on the following three areas: standardised reporting and data collection for diabetic osteomyelitis radiography, standardisation of the scoring system and methodology for diagnosis, and finally larger, more robust studies.

While this review suggests that serial radiography has limited advantages over singular radiography in the diagnosis and monitoring of diabetic foot osteomyelitis, further studies are needed to understand its true clinical utility. Given that this imaging modality is quick, inexpensive, and widely accessible, further research on the value of serial plain radiography is warranted.

## Funding

The authors have nothing to report.

## Conflicts of Interest

The authors declare no conflicts of interest.

## Data Availability

All data analysed in this review were extracted from previously published studies and are publicly available in the cited literature. No new datasets were generated or analysed during the current study.

## Appendix A

**TABLE A1:** Summary of data extraction.

First author	Year of pub.	Country	Study design	Radiologist^a^	Mean Age years (SD)	Male %	Reference standard	*N*	*n*	Sensitivity	Specificity
Serial radiography:											
Leone [22]	2022	Italy	RS	2 (30.3)	57.3 (13.6)	70%	MRI	46	46	89%	38%
Singular radiograph											
Wang [23]	1990	USA	PS	N/A	49	70%	Biopsy	50	46	52%	69%
Morales [24]	2010	Spain	PS	N/A	N/A	N/A	Biopsy	132	105	90%	22%
Levine [25]	1994	USA	PS	N/A	51.6	44%	Biopsy	27	26	60%	81%
Larcos [26]	1991	USA	RS	2 (N/A)	62	61%	B1	49	14	43%	83%
Aragón‐Sánchez [27]	2010	Spain	PS	N/A	N/A	N/A	Biopsy	338	258	82%	93%
Croll [28]	1996	Canada	PS	N/A	66	70%	B2	27	18	22%	94%
Yuh [29]	1989	USA	PS	4 (N/A)	58.2	N/A	Biopsy	29	24	75%	75%
Enderle [30]	1999	Germany	PS	N/A	60.7 (9.8)	89%	Biopsy	19	14	69%	80%
Lipman [31]	1998	USA	PS	N/A	46	65%	Biopsy	20	15	73%	40%
Seldin [32]	1985	USA	PS	N/A	61	N/A	Biopsy	25	14	93%	50%
Oyen [33]	1992	Netherlands	PS	1 (N/A)	62.1	50%	Biopsy	26	7	57%	79%
Park [34]	1982	USA	RS	1 (N/A)	N/A	N/A	Biopsy	36	21	62%	69%
Blume [35]	1997	USA	PS	N/A	64.7	96%	Biopsy	27	20	55%	57%

Abbreviations: B1, biopsy—culture or clinical follow up; B2, biopsy or successful response to medical management; *n*, ⨁ osteomyelitis on reference standard; *N*, total patient sample; PS, prospective study; RS, retrospective study.

^a^ Number of radiologist's evaluating imagery and years of experience.

**TABLE A2:** QUADAS‐2 risk of bias overview.

Study	Risk of bias domains				Applicability concerns		
	Patient selection	Index test	Reference standard	Flow and timing	Patient selection	Index test	Reference standard
Leone [22]							
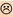
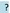
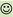
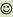
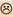
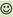
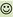
Wang [23]							
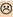
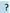
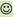
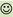
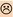
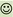
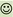
Morales [24]							
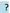
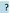
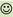
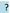
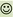
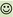
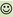
Levine [25]							
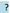
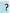
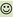
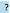
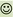
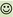
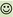
Larcos [26]							
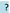
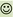
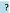
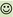
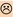
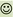
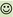
Aragón‐Sánchez [27]							
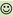
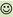
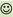
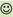
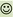
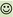
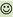
Croll [28]							
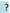
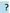
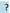
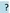
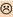
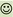
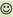
Yuh [29]							
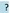
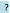
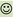
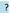
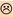
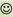
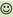
Enderle [30]							
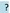
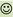
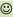
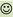
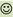
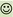
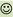
Lipman [31]							
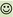
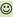
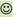
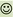
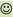
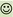
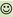
Seldin [32]							
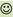
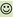
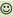
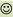
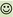
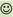
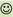
Oyen [33]							
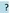
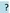
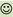
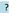
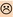
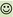
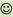
Park [34]							
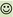
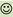
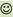
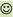
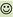
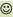
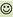
Blume [35]							
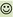
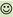
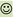
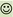
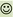
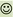
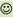

*Note:* : low risk; : high risk; : unclear risk.

**TABLE A3:** Summary of analysis.

First author	Sensitivity			Specificity		
	Sensitivity	*p**	Significant	Specificity	*p**	Significant
Serial radiography						
Leone [22]	89%	1.000	NA	38%	1.000	NA
Singular radiograph						
Wang [23]	52%	0.000	Yes ↓	69%	0.003	No
Morales [24]	90%	0.852	No	22%	0.041	No
Levine [25]	60%	0.004	No	81%	0.000	Yes ↑
Larcos [26]	43%	0.000	Yes ↓	83%	0.003	No
Aragón‐Sánchez [27]	82%	0.244	No	93%	0.000	Yes ↑
Croll [28]	22%	0.000	Yes ↓	94%	0.000	Yes ↑
Yuh [29]	75%	0.127	No	75%	0.003	No
Enderle [30]	69%	0.071	No	80%	0.006	No
Lipman [31]	73%	0.131	No	40%	0.890	No
Seldin [32]	93%	0.663	No	50%	0.424	No
Oyen [33]	57%	0.028	No	79%	0.041	No
Park [34]	62%	0.010	No	69%	0.018	No
Blume [35]	55%	0.002	No	57%	0.153	No

*Note: p* value significance given to three significant figures; arrows indicate direction of significance, **a* ≈ 0.0019.
